# Doctors

**Published:** 1897-09

**Authors:** 


DOCTORS.—We see by a list published in *Virginia Medical Semi-Monthly*, that there are in Texas 4,617 doctors to 2,235,-523 population, or one doctor to every 484 people. [In California there is one to every 383. District of Columbia one to every 264. Whereas, in North Carolina there is one to every 1,191 inhabitants. Mississippi, one to each 943. Georgia one to 909.] Thus it will be seen that Texas is already over stocked with doctors. This alone demonstrates the necessity of more medical colleges in Texas. With one doctor to every 484 people and two schools continually grinding out more, to say nothing of the immigration from other States, is it any wonder that we have quacks galore? There is, so much competition that two-thirds af the graduates have to get at something else, or resort to "ways that are dark and tricks that are vain."

And yet, teachers hold out brilliant futures for those who enter our noble profession. Let some of the plowboys or others who after hearing Professor Dingo ("of European reputation") hold forth on the beauties and pleasures of a practicing physicians life, think of studying medicine, read the paper of Dr. Kinney, published elsewhere in this issue. Dr. Kinney tells a plain unvarnished tale, and his experience tallies with that of thousands of other young men who have been lured into the halls of learning like the moth to the flame, to find only too late that they have been lured to their destruction. If two-thirds of the graduates every year would immediately go to work, and pocket their four years study as so much loss of time it would be better for them and perhaps better for the public, for they cannot all make a living by the legitimate practice, and may have to give it up, sooner or later, or turn quack. Let us have more schools, by all means.

---

THE VACANT CHAIRS in the Texas Medical College announced in our last issue, occasioned by the resignation of Drs. West and Clopton, have not yet been filled. The fall session will open October 1, and it is important that these chairs shall not be vacant. The JOURNAL is not informed as to how many applications there are for these places, nor do we know who are applying. We think, however, that applications from Texas physicians should receive favorable consideration, and as suitable teachers can be found at home we insist that preference should be given them, all things being equal. From conversation and correspondence with numerous members of the profession we are satisfied that this is the prevailing sentiment. It is a slam at our Texas profession to send abroad for teachers.

